# Leptin/Adiponectin Ratio as a new multidimensional biomarker in obese patients with liver steatosis undergoing VLEKT: results from a pilot study

**DOI:** 10.3389/fnut.2026.1754299

**Published:** 2026-03-20

**Authors:** Francesco Balestra, Rossella Donghia, Maria De Luca, Dolores Stabile, Sergio Coletta, Giorgia Panzetta, Rita Palieri, Martina Di Chito, Giovanni De Pergola, Gianluigi Giannelli, Pasqua Letizia Pesole, Maria Principia Scavo

**Affiliations:** 1 Laboratory of Molecular Medicine, National Institute of Gastroenterology IRCCS “S. de Bellis”, Research Hospital, Bari, Italy; 2 Data Science Unit, National Institute of Gastroenterology IRCCS “S. de Bellis”, Research Hospital, Bari, Italy; 3 Core Facility Biobank, National Institute of Gastroenterology IRCCS “S. de Bellis”, Research Hospital, Bari, Italy; 4 Center of Nutrition for the Research and the Care of Obesity and Metabolic Diseases, National Institute of Gastroenterology IRCCS “Saverio de Bellis”, Bari, Italy; 5 Scientific Direction of National Institute of Gastroenterology IRCCS “De Bellis”, Bari, Italy

**Keywords:** HOMA, LAR, liver fibrosis, obese patients, VLCKD, VLEKT

## Abstract

**Introduction:**

The Very-Low Energy-Ketogenic Therapy: (VLEKT) is an effective therapy for obesity and metabolic dysfunction, but the factors driving variability in the treatment response remain unclear. HOMA is a well-established marker of insulin sensitivity during dietary interventions, whereas the Leptin/Adiponectin Ratio (LAR) appears as a novel indicator of adipose tissue inflammation and endocrine remodeling.

**Methods:**

Thirty-seven adults with obesity completed an 8-week VLEKT. Anthropometry, body composition, liver status (FibroScan®), serum biochemistry, circulating adipokine and fibrogenic markers (leptin, adiponectin, resistin, chemerin, visfatin, RBP4, SHBG, FGF21, PAI-1, and follistatin) were assessed at baseline (T0) and post-intervention (T1). LAR or HOMA associations with anthropometric, hepatic, renal, and inflammatory parameters were analyzed using Spearman correlations.

**Results:**

VLEKT produced significant reductions in body weight, BMI, fat mass, fasting glucose, insulin, HOMA, triglycerides, LDL, CAP, and liver stiffness. LAR decreased markedly, indicating improved adipose endocrine–inflammatory balance, while chemerin and RBP4 also declined significantly. Baseline HOMA predicted dyslipidaemia and hepatic steatosis at T1, and longitudinal changes in HOMA correlated with improvements in BMI, lipid profile, fat mass, and GGT. LAR demonstrated broader systemic associations: higher baseline LAR was linked to lower fat-free mass and impaired renal markers, whereas its reduction correlated with improved steatosis, creatinine, uric acid, and calcium homeostasis.

**Discussion:**

VLEKT induced substantial metabolic and inflammatory remodeling. LAR emerged as a multidimensional biomarker reflecting adipose tissue inflammation, hepatic adaptation, and renal homeostasis, while HOMA primarily captured changes related to insulin sensitivity, lipid metabolism, and hepatic status. Their complementary profiles support combined use for personalized monitoring of VLEKT response and early identification of metabolic improvement.

## 1 Introduction

The Very Low-Energy Ketogenic Therapy (VLEKT), is a nutritional intervention characterized by a stringent reduction of carbohydrate intake (<50 g/day), accompanied by moderate protein consumption and the predominance of lipids as the primary energy source (1–3). VLEKT represents the evolution of the former Very Low-Calorie Ketogenic Diet (VLCKD), embodying a paradigm shift in clinical nutrition that redefines ketogenic intervention from a weight-loss strategy centered on caloric restriction to a targeted metabolic therapy in which nutritional ketosis constitutes the principal mechanistic and therapeutic axis (1). This dietary regimen induces a controlled state of nutritional ketosis, marked by elevated circulating ketone bodies, especially β-hydroxybutyrate (3-HB), which serve as alternative energy substrates and also act as potent metabolic signaling molecules with epigenetic, hormonal, and anti-inflammatory properties (4–6). In contemporary clinical practice, VLEKT is increasingly employed in the management of obesity, insulin resistance, and metabolic syndrome, with accumulating evidence supporting its beneficial effects on related complications such as hepatic dysfunction and fibrosis (7, 8). However, the interplay between systemic metabolism, visceral adiposity, and fibrotic progression is still not fully understood. A central mechanistic axis linking metabolic inflammation and hepatic fibrogenesis is the endocrine-metabolic interaction between adipose tissue and the liver. This interaction is mediated by adipokines such as leptin, adiponectin, resistin, and visfatin (9).

VLEKT is proposed to modulate this hepato-adipose axis by mitigating insulin resistance, reducing visceral fat accumulation, and suppressing low-grade chronic inflammation (10). Nevertheless, clinical outcomes demonstrate heterogeneity: patients with mild fibrosis often benefit markedly from carbohydrate restriction and ketosis, whereas those with advanced fibrosis exhibit persistent metabolic dysregulation and impaired bioenergetic flexibility (11). In seeking to define and integrate the various physiological domains modulated by the VLEKT, current evidence suggests that the metabolic response to VLEKT may be contingent upon mitochondrial adaptability and the liver’s ability to enhance fatty acid oxidation pathways. Key profibrotic adipokines, such as leptin, resistin, chemerin, and retinol-binding protein 4 (RBP4), tend to decrease following ketogenic dietary interventions, indicating attenuated fibrogenic signaling (12, 13). Conversely, protective adipokines and metabolic regulators, including adiponectin, sex hormone-binding globulin (SHBG), and fibroblast growth factor-21 (FGF-21), generally increase, exerting anti-inflammatory and anti-fibrotic effects by modulating pathways such as TGF-β signaling and promoting metabolic homeostasis (13). Similarly, follistatin, a natural antagonist of profibrotic activins, shows elevated expression post-intervention, whereas plasminogen activator inhibitor-1 (PAI-1), a driver of extracellular matrix accumulation, is often reduced (14).

Within this complex regulatory milieu, the leptin to adiponectin ratio (LAR) appears as a pivotal biomarker encapsulating the balance between pro-inflammatory and anti-inflammatory adipokine signaling, integral to metabolic homeostasis. Leptin is produced in proportion to the amount of adipose tissue and acts as a pro-inflammatory adipokine, promoting inflammatory signaling and the activation of immune cells (15). Adiponectin, also secreted by adipocytes, generally has anti-inflammatory effects, and its levels decrease in obesity, correlating with increased inflammation and metabolic dysregulation (16, 17).

Parallel to adipokine-derived markers, insulin resistance is a central component of metabolic dysfunction in obesity and represents an important therapeutic target of VLEKT. The assessment of insulin resistance (HOMA) is widely used as a surrogate measure of the ratio between glucose and systemic insulin and is highly sensitive to carbohydrate restriction (18, 19). VLEKT typically leads to a rapid decrease in fasting glucose and insulin concentrations, resulting in substantial improvements in HOMA that reflect reduced visceral adiposity, attenuated metabolic inflammation, and normalized adipokine signaling (20).

Building on these considerations, this pilot study aims to characterize the systemic metabolic remodeling induced by the VLEKT in obese patients by evaluating endocrine-inflammatory networks, adipokines, fibrogenic markers, and regulatory proteins. This study focuses on the clinical utility of using LAR in association with HOMA. By integrating clinical and biochemical values, it provides preliminary evidence of VLEKT’s therapeutic potential and clarifies how LAR and HOMA can be strategically applied to optimize patient management throughout the spectrum of obesity-related metabolic dysfunction.

## 2 Materials and methods

### 2.1 Study population and clinical-biochemical assessments

This eight-week clinical retrospective study was conducted at the Center of Nutrition for Obesity and Metabolic Diseases, National Institute of Gastroenterology “Saverio De Bellis” Research Hospital (Castellana Grotte, Bari, Italy). Thirty-seven obese adults aged 18–64 years (BMI ≥ 30 kg/m^2^) were enrolled. Exclusion criteria included excessive alcohol intake (>20 g/day women; >30 g/day men), type 1 diabetes mellitus, severe cardiovascular or respiratory diseases, gastrointestinal disorders, chronic kidney disease, psychiatric illnesses, pregnancy or lactation, eating disorders, substance abuse, active infections, and rare metabolic or genetic conditions. Behavioral factors such as smoking and daily alcohol consumption were assessed via standardized structured interviews in accordance with established American and European clinical guidelines. The study protocol obtained ethical approval (approval no. 179/C.E. de Bellis, 13 May 2022) in compliance with the Declaration of Helsinki, and all participants provided informed consent. Recruitment spanned July 2022 to December 2023, with evaluations at baseline (T0) and after 8 weeks (T1) of a VLEKT. The diet provided 650–800 kcal/day, emphasizing lipid intake primarily from extra-virgin olive oil, moderate protein, and strict carbohydrate restriction (<30 g/day). Participants were instructed to hydrate adequately (≥2 L water/day), and micronutrient supplementation was systematically administered to avoid deficiencies. Dietary adherence was monitored by daily food diaries. Anthropometry was performed with calibrated instruments, and body composition was evaluated using bioelectrical impedance analysis (BIA) according to ESPEN guidelines. BIA employed a single-frequency analyzer (50 kHz, BIA-101; Akern Bioresearch, Florence, Italy), with participants measured supine after fasting and abstinence from physical activity, food, and alcohol for at least 12 h. Electrodes were placed following standardized anatomical landmarks, and resistance/reactance values recorded to estimate fat mass, fat-free mass, total body water, and extracellular water. Liver status, including fibrosis and steatosis, was assessed via transient elastography (FibroScan® Expert 630, Echosen Italia SRL, Verona, Italy) after a minimum six-hour fast. Measurements of liver stiffness (kPa) and Controlled Attenuation Parameter (CAP; dB/m) were obtained from the right hepatic lobe. Data quality requirements included acquiring ≥10 valid measurements and an interquartile range less than 30% of the median to ensure reliability.

### 2.2 Biochemical-clinical analysis, and serum protein profiling

Blood samples collected between 8:00 and 9:00 a.m. after overnight fasting were analyzed for fasting serum glucose (FSG), insulin, lipid profile (triglycerides, total cholesterol, LDL-C, HDL-C), liver enzymes (AST, ALT, γGT), uric acid, creatinine, high-sensitivity C-reactive protein, thyroid hormones, and 25-hydroxyvitamin D. Assays utilized the COBAS 8000 autoanalyzer (ROCHE Diagnostic SPA, Monza, Italy), and glycated hemoglobin (HbA1c) was quantified via capillary electrophoresis (Capillarys 3 OCTA system; Sebia Italia S.r.l., Florence, Italy). Insulin resistance was calculated using the HOMA formula (21):


HOMA=(FSG(mg/dL)∗fasting insulin(mIU/mL))/405

The modulation of specific circulating proteins was quantified by enzyme-linked immunosorbent assay (ELISA) on serum of patients, applying manufacturer protocols. The assays targeted key adipokines and fibrogenic markers fundamental to mediating the metabolic-inflammatory crosstalk in obesity-associated liver pathology. Table 1 lists the proteins assayed, catalog numbers (Invitrogen, Vienna, Austria), and assay sensitivities.

**Table 1:** Lists of proteins assayed, catalog numbers, and assay sensitivities.

Protein	Catalog COD#	Limit of detection
Leptin	KAC2281	<0.3 pg/mL
Resistin	BMS2040	3.1 pg/mL
Chemerin	EHRARRES2	0.5 ng/mL
RBP4	BMS2199	0.034 ng/mL
Adiponectin	KHP0041	100 pg/mL
SHBG	EH421RB	1.2 pmol/L
FGF21	EH188RB	8 pg/mL
Follistatin	EHFST	500 pg/mL
PAI-V1	KHC3071	<30 pg/mL
Visfatin	EH482RB	1.1 ng/mL

The LAR is calculated using the following formula:


LAR=Leptin/Adiponectin

### 2.3 Statistical analysis

Patients’ characteristics are reported as median and interquartile range (IQR) for continuous variables, and frequency and percentage (%) for categorical. The Wilcoxon matched-pairs signed-rank test was applied for continuous parameters to evaluate variations after the VLEKT of observation. The Spearman rank correlation coefficient was used to test the strength and direction of the association existing between LAR or HOMA and examined variables (i.e., anthropometric & hematic, renal, liver, circulating adipokines and fibrogenic markers).

Additionally, a retrospective power analysis was performed to confirm that our sample size was sufficient, supporting the reliability of the results.

To test the null hypothesis of non-association, the two-tailed probability level was set at 0.05. The analyses were conducted with StataCorp. 2025. *Stata Statistical Software: Release 19*. College Station, TX: StataCorp LLC., and RStudio (“Cucumberleaf Sunflower” Release).

## 3 Results

### 3.1 Anthropometric and clinical outcomes

A cohort of 37 adult patients who underwent VLEKT was analyzed with parameters assessed both before and after the intervention. The mean age of the participants was 44.00 (18.00) years, and the gender variable was represented by 67.57% women and 32.43% men. Results of the recorded parameters are reported in Table 2. The anthropometric and hematic parameters indicated a significant reduction in body weight (median decrease of 11.00 kg, *p* < 0.0001) and BMI (median decrease of 3.60 kg/m^2^, *p* < 0.0001), demonstrating effective weight loss. Glycemic control improved as evidenced by decreases in fasting blood glucose (5 mg/dL, *p* = 0.0002), insulin levels (6.6 UI/mL, *p* = 0.0002), and the HOMA index (from 4.52 to 2.49, *p* < 0.0001), indicating enhanced insulin sensitivity. HbA1c levels decreased significantly (*p* < 0.0001), supporting improved long-term glycemic regulation. The lipid profile displayed significant improvement with reductions in triglycerides (*p* = 0.0002), LDL cholesterol (*p* = 0.0008), and total cholesterol (*p* < 0.0001), although HDL cholesterol decreased slightly (*p* = 0.0002). Thyroid function parameters showed mixed changes, with a slight decrease in FT3 (*p* = 0.0002) but an increase in FT4 (*p* < 0.0001), while TSH remained stable (*p* = 0.74). Vitamin D levels increased significantly (*p* < 0.0001), indicating improved nutritional status.

**Table 2:** Anthropometric and hematic parameters measured after VLEKT (*n* = 37).

Parameters^*^	Before the diet	After the diet	Δ_After-Before_	*p* ^^^
Weight (Kg)	106.00 (25.00)	95.00 (22.50)	−11.00 (6.00)	<0.0001
BMI (Kg/m^2^)	38.40 (4.90)	34.60 (6.30)	−3.60 (1.85)	<0.0001
Blood Sugar (mg/dL)	94.00 (13.00)	89.00 (13.00)	−5.00 (11.00)	0.0002
Insulin (UI/mL)	18.34 (10.30)	11.10 (9.31)	−6.60 (10.72)	0.0002
HOMA	4.52 (2.61)	2.49 (2.26)	−1.82 (2.98)	<0.0001
HbA1c (mmol/mol)	5.60 (0.40)	5.30 (0.50)	−0.20 (0.40)	<0.0001
Triglycerides (mg/dL)	102.00 (59.00)	79.00 (46.00)	−26.00 (46.00)	0.0002
HDL (mg/dL)	48.00 (12.70)	42.00 (11.90)	−5.00 (10.00)	0.0002
LDL (mg/dL)	123.90 (42.40)	100.70 (42.40)	−31.00 (39.40)	0.0008
Total Cholesterol (mg/dL)	182.00 (44.00)	156.00 (62.00)	−35.00 (43.70)	<0.0001
TSH (mIU/L)	1.76 (1.08)	1.70 (1.03)	0.12 (0.58)	0.74
FT3 (pmol/L)	3.40 (0.49)	2.92 (0.64)	−0.40 (0.54)	0.0002
FT4 (pmol/L)	12.00 (1.80)	13.80 (2.50)	1.50 (2.00)	<0.0001
Vitamin D (IU)	18.80 (6.00)	24.50 (12.60)	3.90 (5.70)	<0.0001

^*^As median and Interquartile Range (IQR). BMI, Body Mass Index; HOMA, Homeostatic Model Assessment; HbA1c, Glycated Hemoglobin; HDL, High-Density Lipoprotein; LDL, Low-Density Lipoprotein; TSH, Thyroid-Stimulating Hormone; FT3, Free Triiodothyronine; FT4, Free Thyroxine; GFR, Glomerular Filtration Rate; PTH, Parathyroid Hormone; CAP, Controlled Attenuation Parameter; FIB-4, Fibrosis-4 Index for Liver Fibrosis; FM, Fat Mass; FFM, Fat Fee Mass; AST, Aspartate Aminotransferase; ALT, Alanine Aminotransferase; GGT, Gamma-Glutamyl Transferase. ^^^Wilcoxon signed-rank test.

In an overview of renal parameters (Table 3), uricemia increased modestly but significantly (*p* = 0.003), while creatinine, glomerular filtration rate (GFR), calcium, phosphorus, and parathyroid hormone (PTH) levels remained stable without significant changes, indicating preserved renal function throughout the ketogenic intervention.

**Table 3:** Renal parameters measured after VLEKT (*n* = 37).

Parameters ^*^	Before the diet	After the diet	Δ_After-Before_	*p* ^^^
Uricemia (mg/dL)	5.50 (2.30)	6.10 (1.60)	0.60 (1.50)	0.003
Creatininemia (mg/dL)	0.78 (0.21)	0.77 (0.22)	0.00 (0.08)	0.85
GFR (mL/min)	90.10 (10.00)	89.55 (11.10)	0.00 (3.00)	0.80
Calcium (mg/dL)	4.65 (0.19)	4.67 (0.27)	0.03 (0.30)	0.72
Phosphorus (mg/dL)	3.00 (0.30)	3.30 (0.70)	0.20 (0.70)	0.58
PTH (pg/mL)	55.20 (22.40)	46.90 (30.90)	−2.20 (29.40)	0.28

^*^As median and Interquartile Range (IQR). BMI, Body Mass Index; HOMA, Homeostatic Model Assessment; HbA1c, Glycated Hemoglobin; HDL, High-Density Lipoprotein; LDL, Low-Density Lipoprotein; TSH, Thyroid-Stimulating Hormone; FT3, Free Triiodothyronine; FT4, Free Thyroxine; GFR, Glomerular Filtration Rate; PTH, Parathyroid Hormone; CAP, Controlled Attenuation Parameter; FIB-4, Fibrosis-4 Index for Liver Fibrosis; FM, Fat Mass; FFM, Fat Fee Mass; AST, Aspartate Aminotransferase; ALT, Alanine Aminotransferase; GGT, Gamma-Glutamyl Transferase. ^^^Wilcoxon signed-rank test.

The parameters associated with hepatic (Table 4) well-being indicated significant improvements after the VLEKT. Specifically, the marked decrease in Controlled Attenuation Parameter (CAP) values reflects a substantial reduction in liver steatosis (*p* < 0.0001), suggesting an amelioration of hepatic fat accumulation. Additionally, liver stiffness (E, kPa) also decreased significantly (*p* < 0.0001), indicating potential amelioration of fibrosis or inflammation. The fibrosis score (FIB-4) showed a borderline increase (*p* = 0.05), which may warrant further monitoring. Significant reductions in fat mass (FM) and fat-free mass (FFM) were noted (*p* < 0.0001 and *p* = 0.006, respectively), reflecting overall body composition changes. The liver enzymes AST and ALT demonstrated slight overall improvement, with ALT showing a slight but significant decrease (*p* = 0.02). In addition, GGT decreased significantly (*p* < 0.0001), indicating improved liver function and reduced hepatic injury.

**Table 4:** Liver parameters measured after VLEKT (*n* = 37).

Parameters^*^	Before the diet	After the diet	Δ_After-Before_	*p* ^^^
CAP (dB/m)	314.00 (71.00)	257.00 (96.00)	−57.00 (58.00)	<0.0001
E (kPa)	6.00 (4.30)	4.90 (1.90)	−1.30 (2.60)	<0.0001
FIB-4	0.59 (0.27)	0.65 (0.30)	0.06 (0.22)	0.05
FM (Kg)	45.90 (17.00)	38.90 (14.10)	−7.00 (5.40)	<0.0001
FFM (Kg)	58.30 (20.91)	55.60 (18.01)	−3.20 (4.26)	0.006
AST (U/L)	18.00 (14.00)	20.00 (11.00)	1.00 (5.00)	0.73
ALT (U/L)	21.00 (32.00)	22.00 (21.00)	−3.00 (8.00)	0.02
GGT (U/L)	22.00 (26.00)	17.00 (12.00)	−5.00 (12.00)	<0.0001

^*^As median and Interquartile Range (IQR). BMI, Body Mass Index; HOMA, Homeostatic Model Assessment; HbA1c, Glycated Hemoglobin; HDL, High-Density Lipoprotein; LDL, Low-Density Lipoprotein; TSH, Thyroid-Stimulating Hormone; FT3, Free Triiodothyronine; FT4, Free Thyroxine; GFR, Glomerular Filtration Rate; PTH, Parathyroid Hormone; CAP, Controlled Attenuation Parameter; FIB-4, Fibrosis-4 Index for Liver Fibrosis; FM, Fat Mass; FFM, Fat Fee Mass; AST, Aspartate Aminotransferase; ALT, Alanine Aminotransferase; GGT, Gamma-Glutamyl Transferase. ^^^Wilcoxon signed-rank test.

### 3.2 Biochemical analysis

#### 3.2.1 Changes in circulating adipokines and fibrogenic markers

The circulating adipokines and fibrogenic parameters, measured using ELISA, following the VLEKT in a cohort of 37 adult patients, exhibited significant biochemical changes pertinent to metabolic and inflammatory status, as detailed in Table 5. These molecular changes likely contribute to the clinically observed improvements seen in insulin sensitivity, lipid metabolism, and inflammatory status.

**Table 5:** Circulating adipokines and fibrogenic parameters after VLEKT (*n* = 37).

Parameters^*^	Before the diet	After the diet	Δ_After-Before_	*p* ^^^
Follistatin (g/L)	15.45 (10.66)	15.78 (12.51)	0.26 (4.24)	0.99
Resistin (ng/mL)	10,060.00 (7,634.00)	10,060.00 (10,322.00)	−262.00 (5,853.00)	0.74
SHBG (nmol/L)	29,730.00 (14,150.00)	41,310.00 (37,990.00)	11,440.00 (21,970.00)	0.001
Chemerin (ng/mL)	21.19 (13.25)	18.15 (6.42)	−4.17 (6.62)	0.002
PAI-1 (ng/mL)	3,430.50 (1,973.00)	3,026.00 (2,313.50)	−309.50 (1,259.50)	0.13
Visfatin (ng/mL)	1.06 (2.66)	0.89 (2.41)	−0.24 (0.55)	0.06
FGF-21 (pg/mL)	107.22 (218.61)	84.82 (209.53)	−14.68 (86.29)	0.19
RBP4 (g/mL)	24.21 (16.76)	18.87 (12.05)	−5.27 (12.26)	0.04
Leptin (ng/mL)	26.34 (19.91)	12.55 (9.92)	−10.91 (10.45)	<0.0001
Adiponectin (ng/mL)	2,670.00 (3,921.20)	2,568.00 (3,598.40)	−23.70 (523.20)	0.02
LAR	0.009 (0.21)	0.005 (0.09)	−0.004 (0.10)	<0.0001

^*^As median and Interquartile Range (IQR). SHBG, Sexual Hormone Binding Globulin; PAI-1, Plasminogen Activator Inhibitor-1; FGF-21, Fibroblast Growth Factor 21; RBP4, Retinol Binding Protein 4; LAR, Leptin-to-Adiponectin Ratio. ^^^Wilcoxon signed-rank test.

SHBG levels significantly increased (*p* = 0.001), which may indicate improved hormonal regulation and metabolic health. Chemerin levels significantly decreased (*p* = 0.002), together with leptin (*p* < 0.0001) and adiponectin (*p* = 0.02), suggesting an attenuation of inflammatory processes accompanied by the decrease in the LAR (*p* < 0.0001). Similarly, RBP4 showed a modest but significant decrease (*p* = 0.04), which may reflect improved insulin sensitivity and reduced metabolic risk. In contrast, resistin, follistatin, PAI-1, visfatin, and FGF-21 showed no statistically significant changes; however, some of these parameters exhibited trends toward reductions.

#### 3.2.2 LAR and HOMA as key markers of metabolic remodeling induced by VLEKT

The HOMA and LAR index, reported in Tables 2, 5, showed a highly significant reduction after the VLEKT (*p* < 0.001). Pearson correlation analyses were conducted to elucidate the associations between the LAR or HOMA and multifaceted metabolic domains encompassing anthropometric, hepatic, renal, circulating adipokines, and fibrogenic markers both before and after the VLEKT. The objective was to determine if baseline (T0) LAR or HOMA levels could predict metabolic and physiological outcomes post-diet (T1) and to assess the dynamic shifts of these indices throughout the intervention.

**Figure 1:**
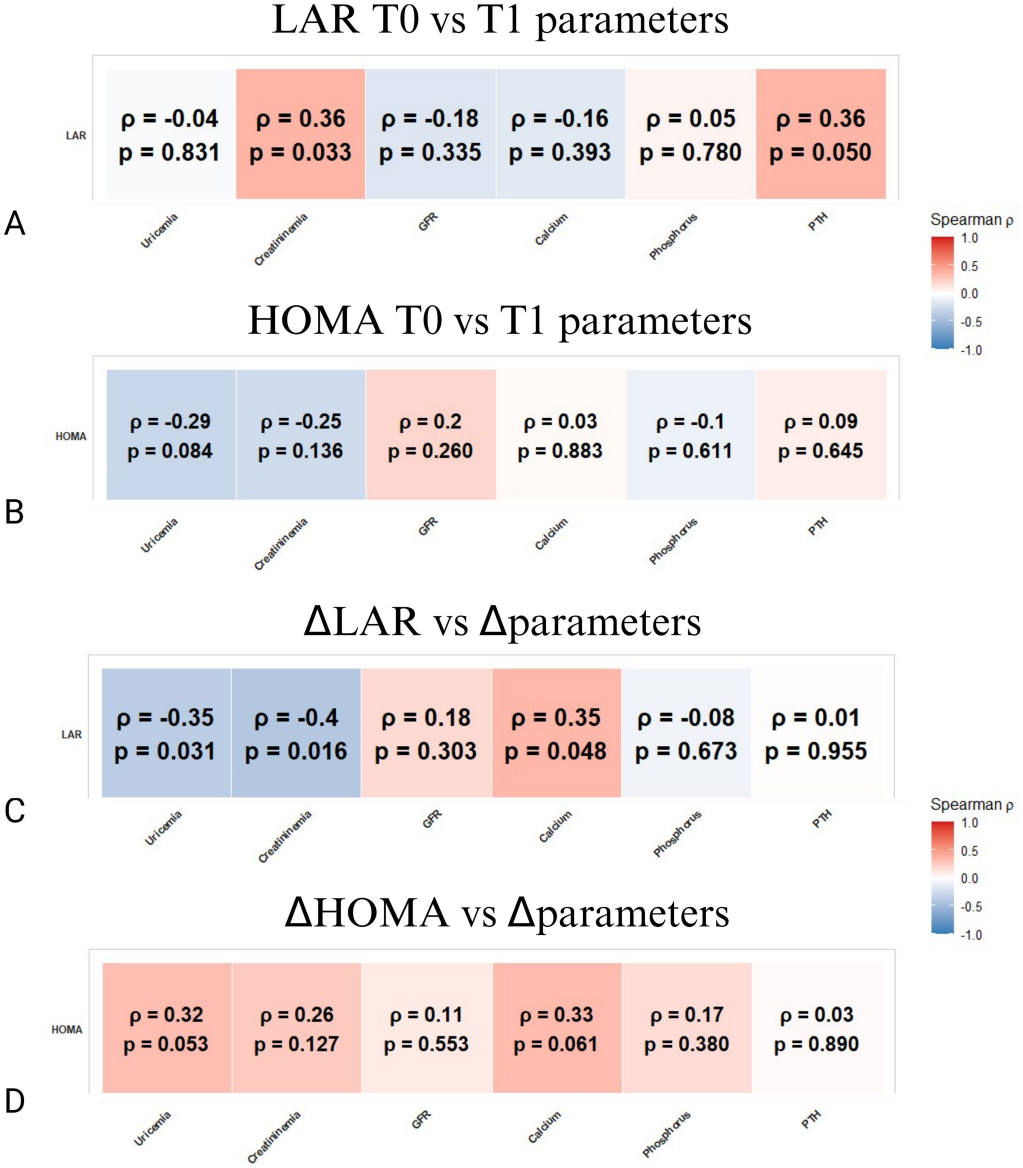
Spearman correlation matrices between LAR or HOMA and anthropometric parameters. **(A)** Correlations between LAR at baseline (T0) and parameters at follow-up (T1). **(B)** Correlations between HOMA at baseline (T0) and parameters at follow-up (T1). **(C)** Correlations between ΔLAR (change from T0 to T1) and changes in the same parameters (Δparameters). **(D)** Correlations between ΔHOMA (change from T0 to T1) and changes in the same parameters (Δparameters). The color bar represents Spearman’s *ρ* (red = positive, blue = negative). *p*-values are shown within each cell; *p*-values < 0.05 were considered significant.

In particular, at baseline, LAR did not demonstrate significant correlation with the modulation of the evaluated anthropometric parameters (Figure 1A). Conversely, baseline HOMA was significantly associated with a dyslipidemic profile at follow-up; specifically, it correlated positively with triglycerides (*ρ* = 0.33, *p* = 0.045) and inversely with HDL cholesterol (*ρ* = −0.38, *p* = 0.020), highlighting insulin resistance as a determinant of adverse lipid metabolism despite dietary interventions (Figure 1B). Longitudinal analysis revealed that changes in LAR did not correspond with alterations in general anthropometric measurements (Figure 1C). In contrast, reductions in HOMA were robustly and positively correlated with improvements in BMI (*ρ* = 0.74, *p* < 0.001), triglycerides (*ρ* = 0.43, *p* = 0.008), LDL cholesterol (*ρ* = 0.44, *p* = 0.007), and total cholesterol (*ρ* = 0.34, *p* = 0.038), evidencing the link between enhanced insulin sensitivity and favorable body composition and lipid metabolism remodeling during VLEKT (Figure 1D).

**Figure 2:**
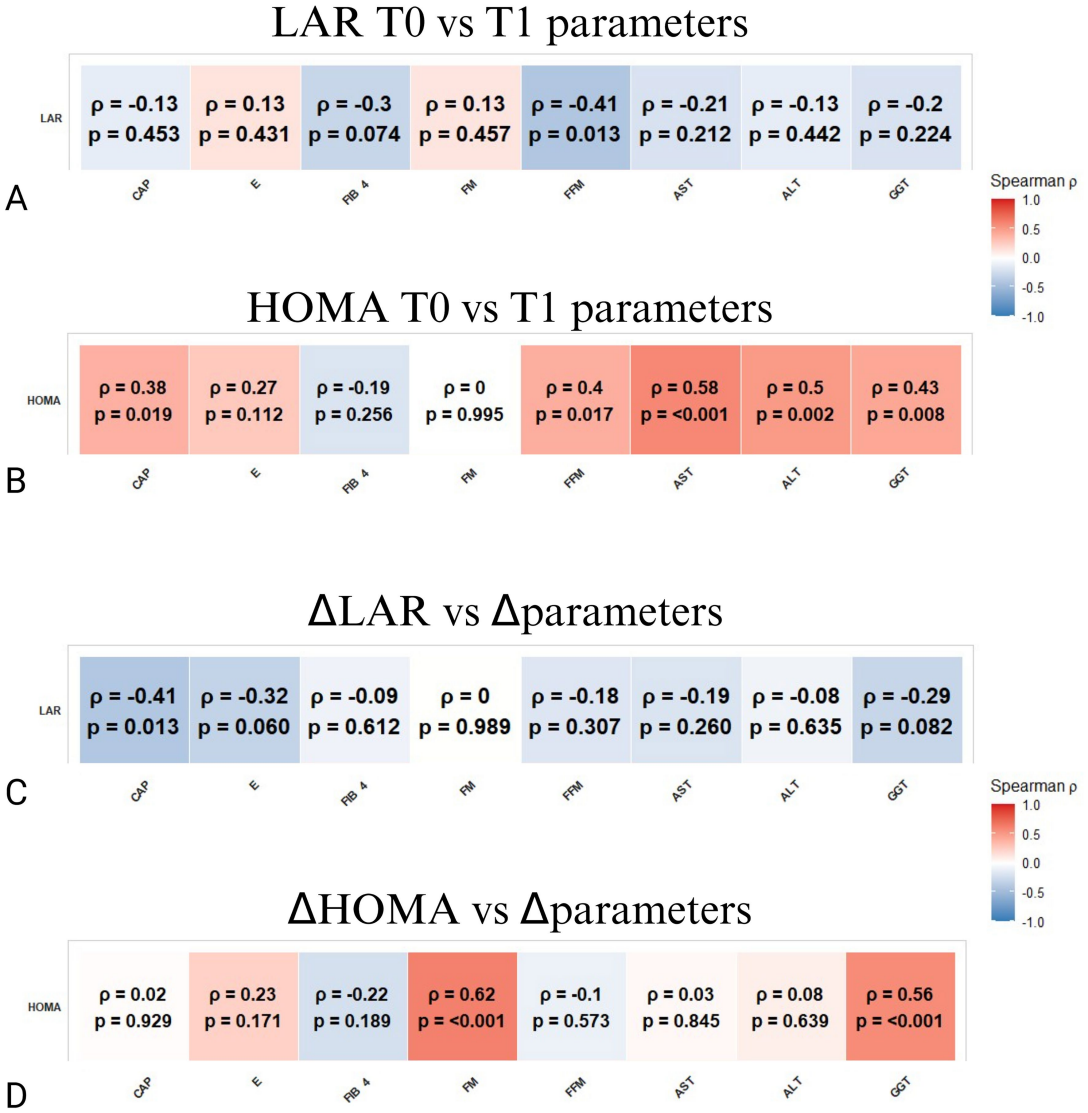
Spearman correlation matrices between LAR or HOMA and hepatic parameters. **(A)** Correlations between LAR at baseline (T0) and parameters at follow-up (T1). **(B)** Correlations between HOMA at baseline (T0) and parameters at follow-up (T1). **(C)** Correlations between ΔLAR (change from T0 to T1) and changes in the same parameters (Δparameters). **(D)** Correlations between ΔHOMA (change from T0 to T1) and changes in the same parameters (Δparameters). The color bar represents Spearman’s *ρ* (red = positive, blue = negative). *p*-values are shown within each cell; *p*-values < 0.05 were considered significant.

Elevated baseline LAR correlated negatively with fat-free mass (FFM) at T1 (*ρ* = −0.41, *p* = 0.013) (Figure 2A), while baseline HOMA demonstrated significant positive associations with hepatic fat content measured by CAP (*ρ* = 0.38, *p* = 0.019) and liver enzymes, including AST, ALT, and GGT (Figure 2B). These findings indicate that individuals with higher insulin resistance exhibited greater hepatic steatosis and elevated liver enzymes following the VLEKT intervention. Dynamic metabolic adaptations were evident as reductions in LAR corresponded with decreases in hepatic steatosis (CAP; *ρ* = −0.41, *p* = 0.013) (Figure 2C), whereas improvements in HOMA correlated with decreases in fat mass (*ρ* = 0.62, *p* < 0.001) and reductions in GGT levels (*ρ* = 0.56, *p* < 0.001), reflecting enhanced insulin sensitivity and liver function recovery (Figure 2D). The decline in LAR reflects enhanced insulin sensitivity, reduced adipose tissue inflammation, and greater metabolic flexibility, consistent with the profound lipid mobilization induced by nutritional ketosis.

Furthermore, renal function analyses revealed that high baseline LAR was positively associated with serum creatinine (*ρ* = 0.36, *p* = 0.033) and parathyroid hormone (PTH) levels (*ρ* = 0.36, *p* = 0.050), markers indicative of compromised renal function and disturbed mineral metabolism.

**Figure 3:**
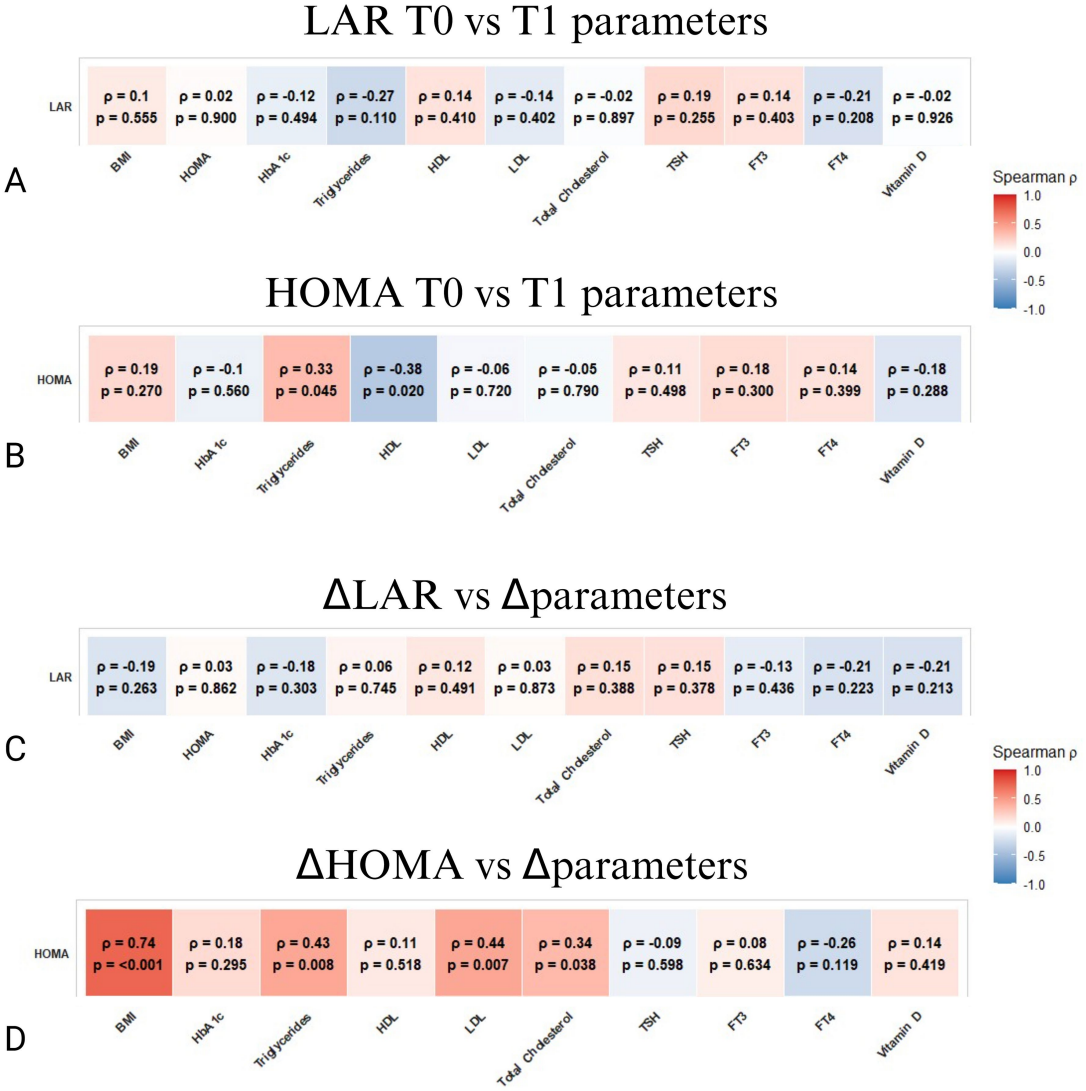
Spearman correlation matrices between LAR or HOMA and the renal parameters. **(A)** Correlations between LAR at baseline (T0) and parameters at follow-up (T1). **(B)** Correlations between HOMA at baseline (T0) and parameters at follow-up (T1). **(C)** Correlations between ΔLAR (change from T0 to T1) and changes in the same parameters (Δparameters). **(D)** Correlations between ΔHOMA (change from T0 to T1) and changes in the same parameters (Δparameters). The color bar represents Spearman’s *ρ* (red = positive, blue = negative). *p*-values are shown within each cell; *p*-values < 0.05 were considered significant.

Notably, LAR reductions were linked to improvements in renal biomarkers, evidenced by negative correlations with serum uric acid (*ρ* = −0.35, *p* = 0.031) and creatinine (*ρ* = −0.40, *p* = 0.016), and a positive correlation with serum calcium (*ρ* = 0.35, *p* = 0.048), underscoring renal and mineral metabolism recovery during VLEKT (Figure 3A).

No significant associations were noted between HOMA and renal parameters (Figure 3B). Overall, the inverse relationship between the reduction in LAR and the increase in these biochemical parameters suggests that LAR may serve as a sensitive predictive biomarker of adipose tissue remodeling and improved metabolic efficiency, even in the presence of short-term renal and mineral homeostatic adjustments typical of the ketogenic transition (Figures 3C,D).

**Figure 4:**
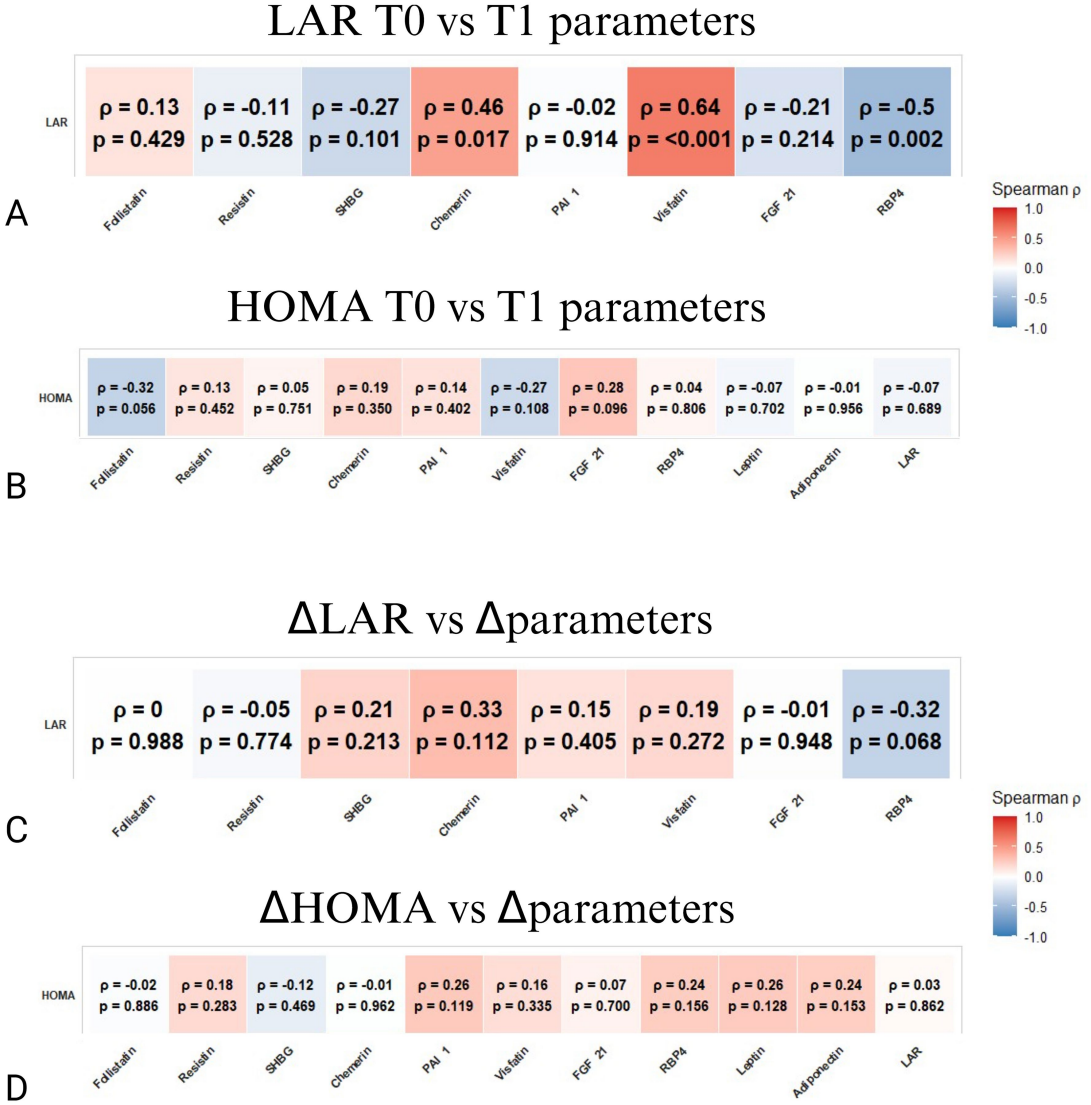
Spearman correlation matrices between LAR or HOMA and the tested circulating adipokines and fibrogenic parameters. **(A)** Correlations between LAR at baseline (T0) and parameters at follow-up (T1). **(B)** Correlations between HOMA at baseline (T0) and parameters at follow-up (T1). **(C)** Correlations between ΔLAR (change from T0 to T1) and changes in the same parameters (Δparameters). **(D)** Correlations between ΔHOMA (change from T0 to T1) and changes in the same parameters (Δparameters). The color bar represents Spearman’s *ρ* (red = positive, blue = negative). *p*-values are shown within each cell; *p*-values < 0.05 were considered significant.

Inflammatory profiling revealed that a higher baseline LAR correlated with elevated circulating levels of the pro-inflammatory adipokines chemerin (*ρ* = 0.46, *p* = 0.017), visfatin (*ρ* = 0.64, *p* = 0.001), linking the leptin/adiponectin imbalance to a pro-inflammatory state, and RPB4 (*ρ* = 0.5, *p* = 0.002) (Figure 4A). Conversely, HOMA demonstrated no significant associations with these inflammatory mediators either at baseline or longitudinally (*p* > 0.05; Figure 4B). Similarly, when analyzing temporal changes (Figures 4C,D), neither ΔLAR nor ΔHOMA showed statistically significant associations with changes in circulating adipokines and fibrogenic parameters (*p* > 0.05).

## 4 Discussion

Monitoring individuals undergoing VLEKT necessitates a comprehensive assessment framework that extends beyond conventional metabolic markers. The inclusion of a wider range of clinical, biochemical, and anthropometric parameters is essential for effective follow-up and individualized management of metabolic health. Such an integrative approach allows for a more accurate evaluation of therapeutic efficacy and long-term outcomes in obesity and related metabolic disorders.

In this pilot study involving 37 participants who underwent an eight-week VLEKT, significant improvements were observed in anthropometric and metabolic indices between T0 and T1. These findings are consistent with evidence reported in previous studies (22–24). The primary aim was to investigate the behavior of the LAR and to compare its information content with that of the well-established HOMA, a validated marker of insulin resistance that is widely used in metabolic research and dietary intervention assessments (25, 26). Results demonstrated a robust reduction in body weight, BMI, fasting glucose, insulin levels, and HOMA, alongside improvements in HbA1c and lipid profiles, involving decreases in triglycerides, LDL cholesterol, and total cholesterol. Liver parameters, including CAP and liver stiffness, also displayed significant improvements, indicating an amelioration of hepatic steatosis and fibrosis, thus corroborating the hepatoprotective effect of VLEKT in obese patients with metabolic complications (27). These results on the relationship between LAR and fibrosis are in line with our previous studies showing that liver fibrosis is positively and independently associated with leptin circulating levels in individuals that are overweight and obese (14).

Of particular interest is the behavior of LAR, which exhibited a pronounced decrease following the ketogenic intervention. This change, primarily driven by leptin reduction and a modest decline in adiponectin, signals a shift in the endocrine and inflammatory status of adipose tissue that is more sensitive than single adipokine assessments. Such findings are in line with studies associating LAR with systemic inflammation, metabolic disarray, and renal function deterioration (28–30). Conversely, HOMA primarily reflected quantitative changes in insulin sensitivity, correlating significantly with lipid parameters, body composition, and hepatic fat content, confirming its reliability in monitoring metabolic adaptation during VLEKT (31).

Correlation analysis highlighted the complementary nature of these two indices: reductions in HOMA were strongly associated with improvements in BMI, lipid profile, and hepatic steatosis, whereas changes in LAR correlated with shifts in inflammatory markers, renal parameters, and systemic metabolic resilience. Furthermore, the T0 vs. T1 correlations reinforce the robust predictive capacity of HOMA, which was consistently linked to a more adverse lipid and hepatic phenotype, reflecting its well-established role as a marker of insulin resistance and metabolic dysfunction. Although certain associations emerged between LAR and renal-related parameters such as creatinine and PTH, these findings should not be interpreted as evidence of renal pathophysiology. Notably, renal function remained clinically stable throughout the intervention, with no significant changes in creatinine levels or GFR. Rather than indicating organ impairment, these correlations likely reflect the systemic metabolic reprogramming induced by VLEKT. Nutritional ketosis is known to transiently influence nitrogen balance, uric acid handling, and mineral homeostasis during the adaptive phase. In this framework, LAR appears to function as a multidimensional integrative biomarker, capturing coordinated endocrine–metabolic adaptations across adipose tissue, liver, and renal axes. Therefore, its association with renal and mineral parameters may signify adaptive cross-talk within the metabolic network rather than long-term pathological alteration. Leptin reduction is expected to parallel fat mass loss, the absence of strong correlations between ΔLAR and anthropometric parameters suggests that LAR may capture adipose tissue functional remodeling beyond simple quantitative fat reduction. However, given the limited sample size, multivariable analyses adjusting for fat mass were not performed, and independence from body composition cannot be definitively established. Dynamic analyses, Δ vs. Δ, further highlighted that reductions in both HOMA and LAR indices were strongly associated with adaptive metabolic improvements induced by the VLEKT intervention. Specifically, decreases in HOMA paralleled favorable changes in lipid profiles and hepatic enzyme normalization, whereas reductions in LAR correlated with amelioration of hepatic steatosis, enhancement of renal function, and restoration of calcium homeostasis.

## 5 Conclusion and limits of the study

Collectively, these findings support the role of LAR as a multidimensional biomarker of systemic metabolic reprogramming during VLEKT, reflecting inflammatory and endocrine axes not fully encompassed by HOMA alone. The marked reduction in LAR observed after the intervention indicates a shift toward improved adipose tissue endocrine balance and decreased inflammatory burden, while the concurrent decrease in HOMA confirms enhanced insulin sensitivity and metabolic flexibility. The complementary association profiles of LAR and HOMA reinforce the clinical value of their combined assessment, enabling a more comprehensive evaluation of therapeutic response and potentially facilitating personalized dietary monitoring in obesity-related metabolic dysfunction. Nevertheless, several limitations must be acknowledged. The relatively small sample size (*n* = 37) and the single-center pilot design limit statistical power and restrict the generalizability of the findings to broader and more heterogeneous populations. Although significant correlations were identified, multivariable analyses adjusting for potential confounding factors, including sex distribution and changes in body composition, were not performed due to sample size constraints. Therefore, larger prospective, randomized, multicenter studies with extended follow-up are warranted to validate these preliminary observations and to more precisely define the predictive and clinical utility of LAR as a routine biomarker in VLEKT management. Another limitation of this study is the absence of dual-energy X-ray absorptiometry (DEXA) for body composition assessment. Instead, we relied on validated anthropometric measures and bioelectrical impedance analysis (BIA), which, although practical and suitable for repeated short-term measurements, are less precise than DEXA. Logistical constraints, cost, equipment availability, and the desire to avoid repeated radiation exposure in healthy participants influenced this decision.

In conclusion, while exploratory in nature, this study provides novel evidence supporting the integrated use of LAR and HOMA as complementary tools for monitoring metabolic remodeling during ketogenic dietary therapy, thereby contributing to the advancement of precision nutrition strategies in obesity and related metabolic disorders.

Finally, an integrated summary of the significant correlations across all metabolic domains is presented in Table 6, accentuating the distinct yet complementary association profiles of LAR and HOMA observed throughout the study.

**Table 6:** Significant Spearman’s correlations between LAR or HOMA, and the main parameter categories, together with the direction of association and their potential physiological implications during the VLEKT intervention.

Parameters category	Index & type of analysis	Parameter(s)	Direction	Potential physiological implication
Anthropometric & hematic	HOMA – T0 vs. T1	Triglycerides	Positive	Higher T0-HOMA predicted higher triglycerides after VLEKT.
	HOMA – T0 vs. T1	HDL cholesterol	Negative	Higher T0-HOMA predicted lower HDL after VLEKT.
	ΔHOMA – Δ vs. Δ	BMI, Triglycerides, LDL, Total cholesterol	Positive	Reduction in HOMA paralleled improvements in lipid profile and body composition.
Hepatic	LAR – T0 vs. T1	FFM	Negative	Higher T0-LAR predicted lower lean mass after VLEKT.
	HOMA – T0 vs. T1	CAP, FFM, AST, ALT, GGT	Positive	T0-HOMA correlated with hepatic fat and hepatic enzyme levels after VLEKT.
	ΔLAR – Δ vs. Δ	CAP	Negative	Reduction in LAR associated with improvement in hepatic steatosis.
	ΔHOMA – Δ vs. Δ	FM, GGT	Positive	Decrease in HOMA accompanied by reduction in fat mass and an important liver enzyme.
Renal	LAR – T0 vs. T1	Creatinine, PTH	Positive	Higher T0-LAR linked to impaired renal function and altered mineral balance after VLEKT.
	ΔLAR – Δ vs. Δ	Uricemia, Creatinine	Negative	Decline in LAR paralleled improvements in renal parameters.
	ΔLAR – Δ vs. Δ	Calcium	Positive	Reduction in LAR associated with normalization of calcium metabolism.
Circulating adipokines and fibrogenic	LAR – T0 vs. T1	Chemerin, Visfatin	Positive	Higher T0-LAR associated with elevated of some inflammatory adipokines after VLEKT.
	LAR – T0 vs. T1	RBP4	Negative	Higher T0-LAR predicted lower inflammation index after VLEKT.

## Data Availability

The datasets presented in this study can be found in online repositories. The names of the repository/repositories and accession number(s) can be found at: ZENODO, doi: 10.5281/zenodo.17712580.
